# Ethanolic Extract of *Rosa rugosa* Roots and Its Bioactive Compound, Oleamide, Prevented Amyloid β-Induced Oxidative Stress and Improved Behavioral Tests in Mice

**DOI:** 10.3390/ijms26094214

**Published:** 2025-04-29

**Authors:** Chan Kyu Park, Soo Jung Choi, Cho Rong Kim, Hyo Ri Shin, Eui-Cheol Shin, Young Jun Kim, Tae Jin Cho, Dong-Hoon Shin, Jae Kyeom Kim

**Affiliations:** 1 Department of Food and Biotechnology, Korea University, Sejong 30019, Republic of Korea; ggoggo382@korea.ac.kr (C.K.P.); rong111@naver.com (C.R.K.); tlsgyfl0524@korea.ac.kr (H.R.S.); yk46@korea.ac.kr (Y.J.K.); microcho@korea.ac.kr (T.J.C.); 2 Novel Food Division, Food Safety Evaluation Department, National Institute of Food & Drug Safety Evaluation, Cheongju-si 28159, Republic of Korea; sjchoi78@korea.kr; 3 Department of GreenBio Science, Gyeongsang National University, Jinju 52725, Republic of Korea; eshin@gnu.ac.kr; 4 Department of Health Behavior and Nutrition Sciences, University of Delaware, Newark, DE 19711, USA

**Keywords:** amyloid β, cognitive impairment, oleamide, oxidative stress, *Rosa rugosa* roots

## Abstract

Researchers have long focused on the accumulation of amyloid beta (Aβ) peptides in the brain as a primary pathological hallmark driving cognitive decline. This study investigated the neuroprotective effects of *Rosa rugosa* (RR) root extract and its key bioactive constituent, oleamide, against amyloid beta (Aβ)-induced neurotoxicity. Initially, an ethanolic extract of RR root was screened via in vitro assays to assess antioxidant and cytoprotective potential in rat pheochromocytoma cells. Subsequent fractionation, open-column chromatography, and preparatory thin-layer chromatography led to the isolation of oleamide, confirmed by gas chromatography–mass spectrometry and ^1^H/^13^C nuclear magnetic resonance analyses. In vivo experiments using intracerebroventricularly injected Aβ in male mice demonstrated that both RR root extract and oleamide significantly improved cognitive performance in the Y-maze and passive avoidance tests. Additionally, oleamide restored acetylcholine levels and reduced malondialdehyde concentrations in brain tissue, indicating mitigation of oxidative stress and support of cholinergic function. No significant toxicity was observed, as evidenced by stable serum transaminase levels and unaltered body or brain weights. These findings highlight oleamide’s potential to protect against Aβ-driven pathology through multiple mechanisms, including reduced lipid peroxidation and improved neurotransmission. Further investigations into oleamide’s molecular targets and synergy with existing therapies may advance its development as a novel candidate for Alzheimer’s disease prevention or adjunct treatment.

## 1. Introduction

Over the past few decades, dementia—particularly Alzheimer’s disease (AD)—has drawn increasing attention due to its rising global incidence and the significant socioeconomic burden it imposes. Researchers have long focused on the accumulation of amyloid beta (Aβ) peptides in the brain as a primary pathological hallmark driving cognitive decline in AD [1]. However, this field recently faced a major controversy following allegations of data manipulation in key Aβ-related studies. A significant scandal emerged in 2022 when an investigation revealed potential image fabrication in a high-profile study that claimed that a specific oligomeric form of Aβ (Aβ*56) was responsible for memory impairment. This revelation raised serious concerns about the integrity of foundational research supporting the amyloid hypothesis. Despite these controversies, the debate over Aβ’s role in AD pathogenesis remains active. While the scandal prompted renewed scrutiny of Aβ-centric hypotheses, it does not entirely invalidate the substantial body of evidence linking Aβ pathology to Alzheimer’s progression. Contemporary investigations continue to explore the complex interplay between Aβ, tau proteins, neuroinflammation, and other molecular events to develop a more comprehensive understanding of AD’s multifactorial nature [2].

Despite the development of drugs that have received approval from the U.S. Food and Drug Administration for Alzheimer’s disease, current therapeutics largely focus on symptomatic relief or modestly slowing disease progression, rather than outright prevention [3]. As a result, there remains a strong demand for novel strategies—particularly preventive interventions—that may alter disease trajectory before irreversible neuronal damage occurs. This has spurred a surge of interest in lifestyle modifications and nutritional approaches to support healthy cognitive aging [4]. A growing number of studies are delving into bioactive compounds in foods, with the hope of intercepting AD processes at earlier stages, including studies we have reported elsewhere [5,6].

Within this context, the root of *Rosa rugosa* (RR) is a promising traditional remedy given its rich content of phenolic compounds, vitamins, and other bioactive constituents. RR has demonstrated antioxidant and anti-inflammatory effects in various disease models, ranging from inflammatory disorders to metabolic dysfunction [7]. In traditional East Asian medicine, the root of RR has been utilized for the treatment of various ailments, including inflammatory conditions, dysentery, and menstrual irregularities. These historical applications are documented in classical Korean and Chinese pharmacopeias and are increasingly supported by recent ethnopharmacological studies [8,9]. In particular, RR root extract has demonstrated antioxidant and lipid-lowering effects in 3T3-L1 cells, suggesting that the whole extract possesses physiological activities beyond those of individual compounds [8]. A comprehensive review by Zhang et al. [9] further highlights the broad pharmacological profile of RR, encompassing its anti-inflammatory, antioxidant, and metabolic regulatory properties. These findings reinforce the traditional perception of RR root as a functional remedy and support its potential utility in preventing or mitigating neurodegenerative processes such as those observed in Alzheimer’s disease. Likewise, some studies have uncovered potential protective mechanisms—such as the suppression of oxidative stress—in cellular and animal models, hinting at broader benefits that may extend to age-related neurodegeneration [10]. However, despite these encouraging findings, direct evidence of RR root’s capacity to counteract Aβ-induced neurotoxicity—a core pathological feature of AD—remains scarce. Specifically, few studies have identified or validated the individual constituents in RR root responsible for neuroprotection in the context of Aβ accumulation. One study found that extracts of RR exhibited neuroprotective effects against oxidative stress-induced cognitive dysfunction, suggesting potential implications for AD [11], but did not address whether RR directly modulates Aβ-mediated mechanisms.

Among the various bioactive components in plants with potential central nervous system activity, oleamide has attracted increasing attention. Oleamide is an endogenous fatty acid amide first identified in cerebrospinal fluid and is known to regulate sleep, mood, and neural transmission. Importantly, oleamide has been shown to exert neuroprotective effects in several experimental contexts, including enhancement of choline acetyltransferase activity, modulation of microglial inflammation, and promotion of Aβ clearance via phagocytosis. Additionally, studies suggest that oleamide may shift amyloid precursor protein (APP) processing toward the non-amyloidogenic pathway by activating α-secretase (ADAM10), potentially reducing Aβ generation. Despite these promising findings, the presence of oleamide in RR roots has not been previously reported, and its role in mediating RR’s neuroprotective effects has yet to be explored.

Therefore, the present study aims to bridge these gaps by evaluating the neuroprotective efficacy of RR root extract against Aβ-induced oxidative stress and behavioral impairment, identifying the major active constituent within RR root, and assessing the in vivo effects of the isolated compound—oleamide—on cognitive performance, oxidative stress, and cholinergic function in a mouse model of Aβ toxicity. Through this integrative approach, we seek to establish oleamide as a novel phytochemical candidate with therapeutic relevance in the context of AD prevention and intervention.

## 2. Results and Discussion

### 2.1. Screening for Protective Effect of Plant Extracts Against Oxidative Stress In Vitro

To select the active sample extract against oxidative stress on the PC12 cells, various native edible extracts were screened using the 2′,7′-dichlorofluorescein diacetate (DCF-DA) and 3-(4,5-dimethylthiazol-2-yl)-2,5-diphenyl-tetrazolium bromide (MTT) assays, in which hydrogen peroxide was used to induce oxidative stress. The rationale behind this is based on the Aβ accumulation in the brain of AD patients, which exacerbates reactive oxygen species (ROS) generation and cognitive malfunctions. Among the 26 screened plant extracts, several—including those derived from *Euryale ferox Salisb.* and *Ulmus pumila* L.—exhibited moderate protective effects in one or both assays. However, the extract from RR roots consistently showed the most pronounced reduction in intracellular ROS and the highest restoration of cell viability under oxidative stress. This superior performance across both assays provided strong justification for selecting RR root for further bioactivity-guided fractionation and compound identification (Table 1). Specifically, the DCF-DA assay showed that RR roots extract achieved a 91.5% decrease in intracellular oxidative stress (expressed as a protective effect in Table 1), indicating a strong antioxidative capacity. In parallel, the MTT assay revealed a 121.4% improvement in cell viability relative to the hydrogen peroxide-challenged control, suggesting that the RR root extract effectively mitigated ROS-induced cytotoxicity. These findings highlight the potential of RR root as a promising source of neuroprotective agents against oxidative stress, leading to further fractionization of the RR root extract to identify active constituents.

### 2.2. Identification of Chemical Structure of Active Constituent in the RR Root Extract

**Figure 1:**
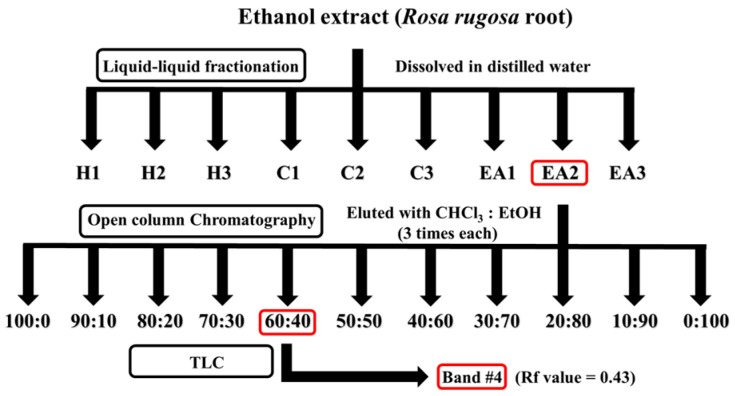
Schematic summary of isolation of protective component from *Rosa rugosa* roots extract. The crude ethanolic extract was subjected to sequential liquid–liquid fractionation using n-hexane (H1–H3), chloroform (C1–C3), and ethyl acetate (EA1–EA3). The most active ethyl acetate sub-fraction (EA2) was further purified using open-column chromatography and preparative thin-layer chromatography (TLC) to isolate the major bioactive compound, oleamide.

**Figure 2:**
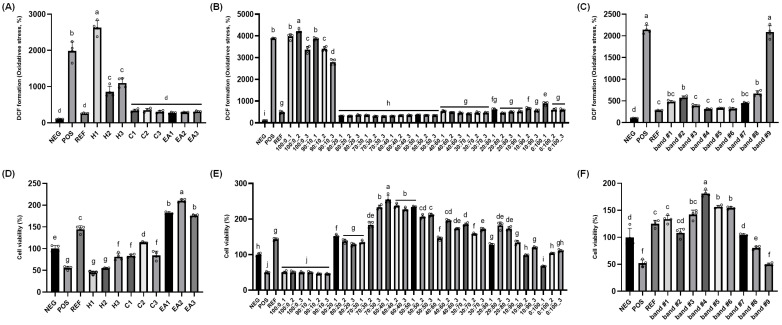
*Rosa rugosa* root extract fractions from liquid–liquid fractionation, silica-gel open column chromatography, and preparatory thin-layer chromatography (TLC) protected rat pheochromocytoma against H_2_O_2_-induced cytotoxicity: Cell viability and intracellular oxidative stress assessments. (**A**–**C**) Intracellular oxidative stress levels were measured using samples from liquid–liquid fractionation, silica-gel open column chromatography, and TLC, (**D**–**F**) Cell viability was measured to examine protective potential of samples from liquid–liquid fractionation, silica-gel open column chromatography, and TLC against oxidative stress. H1–H3: n-hexane fractions; C1–C3: chloroform fractions; EA1–EA3: ethyl acetate fractions; EA2: ethyl acetate fraction #2 selected for further purification; TLC bands 1–9: subfractions separated from EA2 based on Rf value. Normality was assessed using the Shapiro–Wilk test, and the significance was evaluated using one-way ANOVA followed by Dunnett’s post hoc test. Results are shown as means ± standard deviations (*n* = 4). Different superscripts indicate significant differences among groups at *p* < 0.05. Abbreviations: NEG, untreated group; POS, hydrogen peroxide (200 μM) treated group; REF, vitamin C (100 μM) treated group; RR root, *Rosa rugosa* root; TLC, thin-layer chromatography.

Multiple separation and isolation techniques were adopted to identify active constituents responsible for the protective potential of RR root extract (Figure 1). First, the ethanolic extract of RR root was subjected to liquid–liquid fractionization, using *n*-hexane, chloroform, and ethyl acetate, respectively. Fractionization using each solvent was repeated three times and then each layer was evaporated under reduced pressure at 39 °C and prepared for the DCF-DA and MTT assays. In the DCF-DA assay, as expected, the positive control (POS; hydrogen peroxide only) had about 20 times higher intracellular ROS levels relative to the negative control (NEG, no treatment); while the reference control (REF; 100 μM vitamin C treatment) showed dramatic protection compared to the POS (Figure 2A). The inhibitory effect of RR root extracts on intracellular ROS formation in the PC12 cells was demonstrated, although there was no difference in protection between chloroform and ethyl acetate fractions (i.e., C1 through C3 and EA1 through EA3; Figure 2A). In the cell viability assay (i.e., MTT), the treatment with 200 μM hydrogen peroxide (i.e., POS) reduced cell viability to approximately half of the untreated control (NEG), indicating a marked cytotoxic effect. Pre-incubation with 100 μM vitamin C (REF) restored viability to a level exceeding 100% of NEG. Among the tested fractions from RR roots, the hexane (H1–H3) and chloroform (C1–C3) groups exhibited modest protective effects, with viability generally ranging from about 60–90% relative to NEG. In contrast, the ethyl acetate fractions (E1–E3) displayed robust cytoprotection, with E2 in particular elevating viability to roughly 200% of the NEG—surpassing even the REF group (Figure 2B). Based on these results, we selected the EA2 fraction for further purification processes.

Subsequently, the EA2 fraction was subjected to open-column chromatography in which silica gel was used as the stationary phase. Three fractions were collected for each solvent gradient, with a total of 33 fractions collected. Similar to the above, DCF-DA and MTT assays using the PC-12 cells were used. Overall, a pronounced inhibition of DCF formation was observed in fractions eluted with chloroform–ethanol mixtures ranging from 80:20 to 50:50 (Figure 2C). In the MTT assay, the fraction eluted with 60:40 chloroform–ethanol showed the highest cell viability (Figure 2D), and therefore, these fractions were combined and evaporated for TLC analysis. Lastly, the 60:40 chloroform–ethanol fractions were subjected to the TLC plate and then separated. The solvent mixture of chloroform and ethanol (55:45) was employed and the developed bands were visualized using an ultraviolet lamp (245 and 365 nm); each band was scraped, extracted with ethanol, and assessed for protective effects (i.e., MTT and DCF-DA assays). Amongst nine TLC bands, band #4 (Rf value = 0.43) showed superior inhibition of DCF formation as well as cell viability (Figure 2E,F).

**Figure 3:**
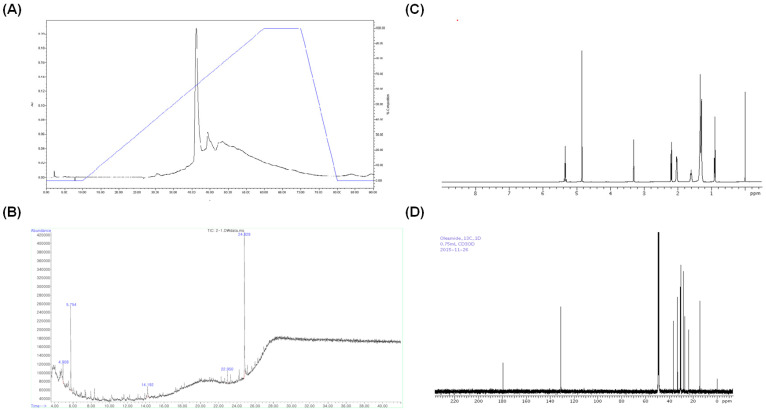
Oleamide from *Rosa rugosa* root extract was chemically characterized using high-performance liquid chromatography (HPLC), gas chromatography–mass spectrometry (GC-MS), and nuclear magnetic resonance (NMR) spectroscopy. (**A**) A representative chromatogram of HPLC for separation of oleamide from *R. rugosa* root extract (retention time of 42 min). The black line represents the compound peaks, while the blue line indicates the mobile phase gradient; (**B**) A representative GC-MS chromatogram of oleamide (retention time of 24.8 min); (**C**) ^13^C-NMR spectrum; (**D**) ^1^H-NMR spectrum.

The selected TLC band was extracted and evaporated under reduced pressure at 39 °C. The obtained sample was dissolved in ethanol and analyzed by HPLC using C_18_ Pak MG S5 column. The data were monitored over the range 200–800 nm, with detection wavelength at 280 nm. A significant peak appeared at 42 min (Figure 3A). GC-MS analysis was subsequently performed by an Agilent 6890 Plus gas chromatograph equipped with a 5977 N mass selective detector quadrupole MS system. As illustrated by the total ion chromatogram (Figure 3B), a prominent peak emerged at around 24.8 min, displaying a mass spectrum consistent with oleamide (oleic acid amide). Following GC–MS analysis of the RR root extract fraction, to confirm this preliminary identification, commercially obtained oleamide was analyzed alongside the isolated fraction using the ^13^C and ^1^H-NMR. As shown, the NMR spectra of the sample fraction and the standard oleamide displayed highly similar chemical shifts and splitting patterns, corroborating that the principal component in the extracted band is indeed oleamide. This combined approach—mass spectrometric matching followed by comparative NMR—provides strong evidence that oleamide is the bioactive constituent isolated from RR root extract (Figure 3C,D).

### 2.3. RR Root Extract and Oleamide Improved Aβ_1-42_-Induced Behavioral Abnormalities and Brain Biomarkers

**Figure 4:**
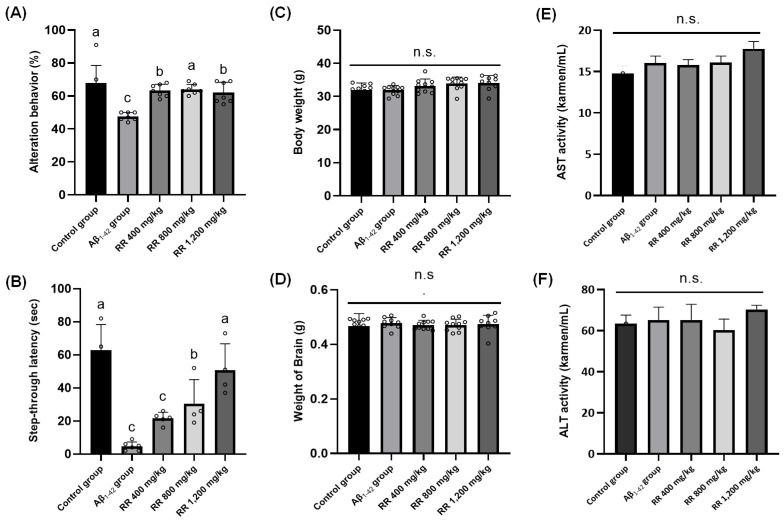
*Rosa rugosa* root extract exhibited neuroprotective effects in an Aβ_1-42_-induced mouse model of cognitive impairment: Y-maze and passive avoidance tests. (**A**) Y-maze test; (**B**) Passive avoidance test; (**C**,**D**) Body and brain weights of mice; (**E**,**F**) AST and ALT activity in the serum of Institute of Cancer Research mice. Control group was injected with Aβ_42-1_, a reverse-sequence peptide of Aβ_1-42_, commonly used as a non-toxic negative control. Aβ_1-42_ group was injected with 410 pmol of Aβ_1-42_ per mouse. Sample groups (RR 400, RR 800, and RR 1200) were injected with 410 pmol of Aβ_1-42_ after being supplemented with extract RR (400 mg/kg, 800 mg/kg, and 1200 mg/kg per day). Normality was assessed using Shapiro–Wilk test, and significance was evaluated using one-way ANOVA followed by Dunnett’s post hoc test. Results are shown as means ± standard deviations (*n* = 7–8). Different superscripts indicate significant differences among groups at *p* < 0.05. Abbreviations: ALT, alanine aminotransferase; AST, aspartate aminotransferase; n.s., not significant; RR, *Rosa rugosa* root.

Two sets of animal intervention studies were conducted to examine the neuroprotective potential of RR root extracts and their bioactive oleamide. First, the percentage of spontaneous alternation in the Y-maze test reflects working memory performance. Mice injected with Aβ_1-42_ (POS) displayed a significantly lower alternation behavior compared to the NEG (*p* < 0.05), indicating impaired spatial working memory induced by the ICV injection in our study. Oral administration of RR root extract at 400, 800, or 1200 mg/kg improved alternation behavior relative to the POS, suggesting a neuroprotective effect. These doses were determined based on previous in vivo studies utilizing plant extracts with similar pharmacological profiles and traditional medicinal use, as reported by Na et al. [11] and Lee et al. [8]. In addition, a study by Park et al. [12] demonstrated that RR root extract exhibited hepatoprotective effects in rats at doses of 250 and 500 mg/kg, supporting the safety and biological activity of this extract in vivo. Accordingly, the 1200 mg/kg dose in our study was included as a high-dose exploratory group to assess potential dose-dependent efficacy and upper-limit tolerability. Notably, no significant toxicity was observed at this high dose, as evidenced by stable body weight, brain weight, and serum transaminase levels, indicating a favorable safety profile. However, no clear dose dependence was observed among the three doses, as each treatment group exhibited statistically comparable improvements over the POS (Figure 4A). In addition, there was no statistical difference between the groups in the number of animals’ arm entries. The improvement in spontaneous alternation performance across all administered doses (400, 800, and 1200 mg/kg) indicates potential memory-enhancing properties. However, the absence of a clear dose–response relationship suggests that the effects may reach a saturation point at lower doses. Figure 4B presents the passive avoidance test results, with step-through latency serving as an index of memory retention. The POS, which received Aβ_1-42_ injection, displayed a markedly lower latency compared to the NEG, indicating impaired avoidance learning. In contrast, mice treated with RR root extract at 400, 800, or 1200 mg/kg showed a noticeable increase in latency relative to the POS group, suggesting a protective effect against Aβ-induced cognitive deficits (Figure 4B). However, again, no clear dose dependence was observed among the three RR treatment groups. Additionally, to monitor any abnormal signs of toxicity, whole body weight, brain weight, as well as serum AST and ALT levels were measured, with no statistical differences noted in any marker (Figure 4C,D).

Although the inclusion of a standard positive control, such as donepezil, tacrine, or *Ginkgo biloba* extract, is commonly employed in behavioral pharmacology studies to contextualize efficacy, we chose not to include such a control in the present work. This decision was based on the well-established reproducibility of Aβ_1-42_-induced cognitive deficits in rodent models, which allowed us to prioritize comparative evaluation between vehicle-treated, Aβ-injected, and test substance-treated groups. Furthermore, at the time the experiments were conducted, *Ginkgo biloba* extract had not been officially recognized as a validated neuroprotective agent by regulatory authorities and thus was not considered an appropriate pharmacological comparator. Nonetheless, we agree that including a recognized positive control would further reinforce the comparative interpretability of our findings. Therefore, future studies should include a standard reference agent to better contextualize the behavioral efficacy of RR root extract and oleamide in comparison to established treatments.

The observed neuroprotective effects of RR root extract align with prior findings demonstrating the antioxidative and anti-inflammatory properties of RR extracts in neurodegenerative models. For instance, RR extract mitigated cognitive dysfunction and stress-induced impairments via serotonergic pathways, suggesting potential benefits for memory-related tasks [11]. Similarly, a study examining RR flower buds identified phenolic glucosides with neuroactive properties that improved sensorimotor gating deficits in mice [13]. While these studies focused on different aspects of cognitive function, they provide supporting evidence that various parts of RR contain bioactive compounds with neuroprotective potential. In line with this, our results provide important insights into the bioactive compound(s) responsible for the protective effects of RR root extract against Aβ_1-42_-induced neurotoxicity in vivo.

**Figure 5:**
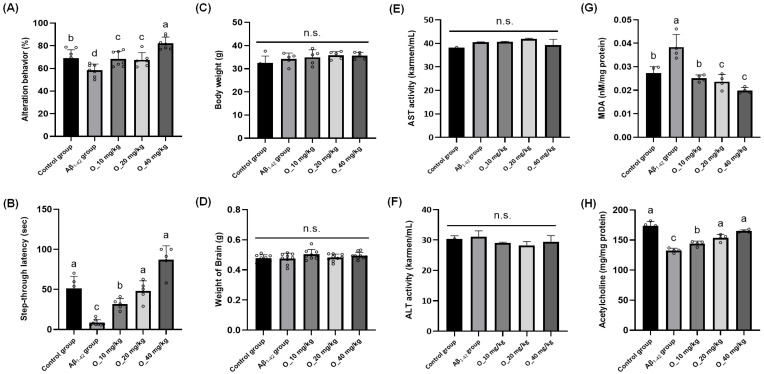
Oleamide showed memory-ameliorating effects against Aβ_1-42_-induced mouse model in behavioral and biochemical analyses. Effects of oleamide supplementation on (**A**) Y-maze test, (**B**) passive avoidance test, (**C**,**D**) body and brain weights of mice, (**E**,**F**) AST and ALT activity in the serum of Institute of Cancer Research mice, and (**G**,**H**) lipid peroxidation (MDA) and acetylcholine (ACh) contents in mice brain tissues. Control group was injected with Aβ_42-1_, a reverse-sequence peptide of Aβ_1-42_, commonly used as a non-toxic negative control. Aβ_1-42_ group was injected with 410 pmol of Aβ_1-42_ per mouse. Sample groups (O_10, O_20, and O_40) were injected with 410 pmol of Aβ_1-42_ followed by feeding with the oleamide (O_10, O_20, and O_40 mg/kg per day). Normality was assessed using Shapiro–Wilk test, and significance was evaluated using one-way ANOVA followed by Dunnett’s post hoc test. Results are shown as means ± standard deviations (*n* = 7–8). Different superscripts indicate significant differences among groups at *p* < 0.05. Abbreviations: ACh, acetylcholine; ALT, alanine aminotransferase; AST, aspartate aminotransferase; MDA, malondialdehyde; RR, *Rosa rugosa* root; n.s., not significant.

To this end, we executed an intervention study with an identical study design using oleamide. Spontaneous alternation behavior in the Y-maze test was evaluated following oleamide treatment at 0, 10, 20, and 40 mg/kg [14]. Relative to the NEG, the Aβ_1-42_-injected group (i.e., POS) displayed a significant decline in alternation percentage, whereas administration of oleamide at 40 mg/kg improved alternation behavior; the two low doses, 10 mg/kg and 20 mg/kg, produced no difference compared to the NEG [Figure 5A]. Step-through latency in the passive avoidance task was substantially reduced in the Aβ_1-42_-injected group (POS) compared to the NEG, signifying impaired memory retention. Treatment with oleamide at 10 mg/kg and 20 mg/kg partially restored latency, and 40 mg/kg elicited greater improvements in the test [Figure 5B]. Again, to monitor the potential toxicity of oleamide intervention, whole body weight, brain weight, as well as serum AST and ALT levels were measured; however, no statistical differences were found [Figure 5C,D]. Additionally, oxidative stress induced by ICV injection of Aβ was assessed through MDA measurements in brain tissue homogenates. As expected, the Aβ-injected group (POS) showed a marked elevation in MDA compared to the NEG, indicating heightened lipid peroxidation. Administration of oleamide produced significant reductions in MDA relative to POS at all levels [Figure 5E]. Lastly, ACh levels in brain tissues were quantified, given that a well-known feature of Aβ-induced neurotoxicity is the reduction of ACh, a critical neurotransmitter for learning and memory [15]. In the present study, Aβ-injected mice (POS) exhibited significantly lower ACh concentrations in the brain compared to the NEG, which aligns with the cholinergic hypothesis of AD, in which Aβ accumulation correlates with the deterioration of cholinergic neurons. In our study, administration of oleamide at 20 mg/kg and 40 mg/kg led to progressively higher concentrations, indicating a dose-dependent improvement. The 40 mg/kg dose in particular returned ACh to near-NEG levels, suggesting that oleamide can help preserve or recover cholinergic function under Aβ-induced stress. In this study, Aβ_1-42_ injection led to elevated levels of malondialdehyde (MDA) and decreased acetylcholine concentrations in mouse brain tissue, indicating significant oxidative stress and cholinergic impairment. Both the RR root extract and its major constituent oleamide effectively reversed these effects, which suggests that the primary neuroprotective mechanism involves the mitigation of Aβ-induced oxidative damage. These findings align with previous reports showing that Aβ accumulation exacerbates reactive oxygen species (ROS) production, leading to lipid peroxidation and disruption of neuronal membrane integrity. The observed reduction in MDA levels and behavioral improvements further support the antioxidant properties of RR and oleamide in preventing Aβ-mediated neuronal dysfunction. While neuroinflammation is another critical factor in AD progression, our study did not directly assess inflammatory markers; thus, we have limited our discussion to the confirmed antioxidative and cholinergic effects. Overall, our second animal intervention study using oleamide generally suggests that Aβ-induced oxidative stress can be attenuated by oleamide in a dose-dependent manner, with higher doses conferring more pronounced protection against lipid peroxidation and decline in ACh concentrations.

While the current study confirmed that oleamide, a major constituent of RR root extract, effectively reduced lipid peroxidation and restored acetylcholine levels in Aβ_1-42_-injected mice, it is important to note that these biochemical assays were not conducted for the RR extract-treated groups. This was primarily due to limitations in sample availability and the study’s targeted focus on compound-level validation of oleamide. Given that the RR extract also showed behavioral improvements comparable to oleamide, it is possible that other constituents within the extract may contribute to similar antioxidant and cholinergic mechanisms. Future studies should aim to include parallel biochemical assessments in extract-treated animals to evaluate potential synergistic effects and to more fully characterize the multi-component nature of RR root’s neuroprotective actions.

The findings from our second animal intervention study aligned with previous studies investigating oleamide’s role in neuroprotection and its potential therapeutic relevance in AD. Oleamide has been identified as a compound that modulates cholinergic signaling, which is critical for learning and memory. Our group previously reported that oleamide enhances the activity of choline acetyltransferase, an enzyme responsible for ACh synthesis, leading to improved cognitive performance in scopolamine-induced amnesia models [16]. This supports our findings that oleamide treatment at 20 mg/kg and 40 mg/kg restored ACh levels and improved behavioral performance. In addition, studies suggest that oleamide exerts neuroprotective effects by reducing oxidative stress and neuroinflammation. For instance, it was reported that oleamide, a bioactive compound in fermented dairy products, enhances microglial phagocytosis of Aβ aggregates while suppressing neuroinflammation [17], which is in good agreement with our results, where a significant reduction in MDA levels was observed following oleamide administration.

Although this study focused on the antioxidant and cholinergic mechanisms of RR root extract and oleamide, we acknowledge that neuroinflammation is a critical contributor to Aβ_1-42_-induced neuropathology. While previous studies have shown that RR extracts can attenuate inflammation in models of metabolic and inflammatory disease, we did not directly assess neuroinflammatory markers such as TNF-α, IL-1β, or microglial activation in the current study. Future work will incorporate such analyses to clarify whether RR root extract also modulates neuroinflammatory pathways in the context of Aβ-induced toxicity.

Aβ toxicity is a central feature of AD pathogenesis, contributing to synaptic dysfunction, neuroinflammation, and neuronal loss. The present study demonstrates that oleamide from RR root extract exerts protective effects against Aβ-induced neurotoxicity. Previous studies suggest that oleamide may mitigate Aβ-related pathology through a dual mechanism involving suppression of microglial activation and enhancement of the non-amyloidogenic APP processing pathway. Neuroinflammation is a key contributor to AD progression, with activated microglia exacerbating neuronal damage through the release of pro-inflammatory cytokines such as tumor necrosis factor-alpha (TNF-α) [18]. The results indicate that oleamide reduces TNF-α levels in microglial cells, an effect that appears to be mediated through P2Y-type G-protein-coupled receptor signaling. This is particularly relevant given that persistent neuroinflammation has been implicated in impaired Aβ clearance and increased neuronal susceptibility to toxicity.

In addition to its role in neuroinflammation, oleamide appears to influence Aβ production through the regulation of α-secretase activity [19]. A recent report suggests that oleamide enhances the activation of ADAM10, a key α-secretase enzyme responsible for the non-amyloidogenic cleavage of APP. This pathway precludes the formation of Aβ peptides by shifting APP metabolism away from the amyloidogenic pathway, thereby reducing Aβ plaque formation. Given that ADAM10 function is known to decline with disease progression, its pharmacological activation has been proposed as a potential therapeutic strategy for AD. The ability of oleamide to stimulate this pathway may thus provide a direct mechanism for limiting Aβ accumulation.

Moreover, accumulating evidence points to oleamide’s immunomodulatory effects on microglial cells. Microglia, as the primary immune cells of the central nervous system, play a dual role in neurodegeneration: while they help clear Aβ aggregates through phagocytosis, prolonged activation can lead to the release of pro-inflammatory cytokines that exacerbate neuronal damage. Oleamide has been shown to reduce the expression of inflammatory mediators such as TNF-α and IL-1β in activated microglia, potentially via modulation of P2Y-type G-protein-coupled receptor signaling. This anti-inflammatory action may contribute to an enhanced microglial phenotype more conducive to Aβ clearance and neuroprotection. These mechanisms, in tandem with oleamide’s antioxidative effects, may offer a more comprehensive explanation for its neuroprotective properties observed in the current study.

While the current findings highlight oleamide as a promising neuroprotective agent, several limitations warrant further investigation. Future studies should aim to delineate the precise molecular signaling pathways through which oleamide modulates ADAM10 activity and inflammatory responses in the brains (in our case, ICV injection of Aβ_1-42_). Although oleamide was identified as a major compound and further validated for its neuroprotective effects, the extract likely contains other bioactive constituents that may contribute to its overall activity. Future studies will aim to isolate and evaluate additional compounds, including flavonoids and triterpenoids, to further understand the multi-component nature of the extract’s efficacy. Long-term administration studies in in vivo AD models will be necessary to evaluate the sustained efficacy and safety of oleamide-based interventions. Additionally, the potential for oleamide to be used in combination with existing anti-Aβ therapies should be explored to determine whether its effects can be augmented through multimodal treatment strategies. Nonetheless, our study presents several key strengths. First, a systematic and comprehensive approach was employed to identify oleamide as the primary bioactive constituent in RR root extract. Through a combination of fractionation, chromatography, GC-MS, and NMR analyses, we successfully isolated and structurally characterized oleamide as the active neuroprotective compound from the RR root extract. Second, we examined both the crude extract (RR root extract) and the isolated bioactive compound (oleamide) in animal experiments, allowing for a more detailed evaluation of their respective neuroprotective effects. Finally, the study also incorporated biochemical analyses to assess ACh levels, oxidative stress marker (MDA), and serum AST/ALT levels, ensuring a thorough evaluation of oleamide’s efficacy and safety profile. The lack of significant toxicity observed at effective doses suggests a favorable application window, supporting the feasibility of oleamide-based interventions against AD.

## 3. Materials and Methods

### 3.1. Materials

DCF-DA, MTT, dimethyl sulfoxide (DMSO), hydrogen peroxide, and L-ascorbic acid (vitamin C) were obtained from Sigma-Aldrich (St. Louis, MO, USA). Silica gel was purchased from Merck (Darmstadt, Germany), and amyloid beta peptide was sourced from Bachem Holding (Bubendorf, Switzerland). Serum transaminase assay kits were supplied by Asan Pharmaceutical (Seoul, Republic of Korea). Unless otherwise noted, all additional chemicals were of analytical grade.

### 3.2. Cell Culture Conditions

The PC12 cell line (CRL-1721) was obtained from the American Type Culture Collection (ATCC; Manassas, VA, USA). RPMI 1640 medium, donor horse serum, fetal bovine serum (FBS), and antibiotic–antimycotic were purchased from Gibco-Invitrogen (Grand Island, NY, USA). Sodium bicarbonate was sourced from Sigma-Aldrich. Sodium chloride, disodium hydrogen phosphate (sodium hydrogen phosphate anhydrous), and potassium dihydrogen phosphate (potassium phosphate monobasic) were obtained from Junsei Chemical (Tokyo, Japan). Potassium chloride was procured from Showa Chemicals (Tokyo, Japan). PC12 cells were cultured in 100 mm tissue culture dishes in a medium supplemented with 10% heat-inactivated horse serum, 5% fetal bovine serum, and 1% antibiotic–antimycotic. The cells were maintained in an incubator at 37 °C with water saturation and 5% of CO_2_. Cultured dishes were dislodged and passaged when each dish was 80–90% confluent. The medium was changed at least three times a week.

### 3.3. Measurement of Intracellular Oxidative Stress in PC12 Cells

Intracellular oxidative stress was assessed using the DCF-DA assay as described elsewhere [5,20]. Briefly, PC12 cells were plated in 96-well plates at a density of 2.0 × 10^5^ cells/mL (100 μL per well). After seeding, cells were pretreated with each test sample (1 mg/mL) for 48 h, then exposed to 100 μM hydrogen peroxide (freshly prepared) or no treatment for two hours. Following this incubation, 250 μM DCF-DA was applied for 50 min. The conversion of DCF-DA to fluorescent 2′,7′-dichlorofluoroscein was measured using a fluorometer (GENois TECAN; Mannedorf, Switzerland) at an excitation wavelength of 485 nm and an emission wavelength of 535 nm.

### 3.4. Measurement of Cytotoxicity Using MTT Reduction Assay

The cytotoxicity of samples was tested using the conventional MTT reduction assay as reported elsewhere [20]. Briefly, PC12 cells were seeded in 96-well plates at a density of 2.0 × 10^5^ cells per mL (100 μL per well) and allowed to adhere overnight. Following pretreatment with test samples (1 mg/mL) for 48 h, cells were exposed to oxidative stress by adding 200 μM hydrogen peroxide for two hours. After the stress period, 0.25 mg/mL MTT solution was added to each well, and the plates were incubated for an additional three hours at 37 °C in a 5% CO_2_ atmosphere. During this incubation, viable cells reduced MTT to insoluble formazan crystals. After incubation, the medium was removed and 150 μL of DMSO was added to each well to solubilize the formazan. The absorbance was then measured at 570 nm with a reference wavelength of 630 nm using a microplate reader (GENois TECAN). Cell viability was calculated as a percentage relative to control wells that were not exposed to oxidative stress.

### 3.5. Preparation of Plant Extracts for Screening

Twenty-six different dried plant materials were purchased from Gyeong-dong Market, an oriental medicine store (Seoul, Republic of Korea). The scientific names of the samples are provided in Table 1. Each sample was verified for identity based on morphological characteristics and stored at 4 °C prior to extraction. Approximately 50 g of each dried sample was pulverized to a fine powder using a laboratory mill. The powdered material was then extracted with 80% ethanol at a ratio of 1:5 (*w*/*v*), followed by stirring at room temperature for 24 h. The extract was filtered through Whatman No. 42 filter paper, and the residue was re-extracted twice under the same conditions to maximize yield. The combined filtrates were concentrated under reduced pressure at 39 °C using a rotary evaporator. Dried extracts were stored at −20 °C until further use. For screening assays, each extract was dissolved in 5% DMSO to a final concentration of 1 mg/mL.

### 3.6. Isolation and Purification of Active Constituent from RR Root Extract: Liquid–Liquid Fractionation, Open-Column Chromatography, and Preparatory Thin Layer Chromatography (TLC)

Approximately 4 kg of RR roots were extracted with 80% ethanol at a ratio of 1:5 (*w*/*v*), followed by stirring at room temperature for 24 h. The extraction process was repeated to maximize yield. The combined filtrates were then concentrated under reduced pressure, resulting in about 221 g of a crude ethanolic extract. Of this total, 20 g of extract was reserved for further in vivo experimentation. To facilitate subsequent fractionation processes, the extract was dissolved in 1600 mL of water. A sequential liquid–liquid fractionation protocol was then performed; first, three separate volumes (each 4800 mL) of n-hexane were added, with each addition followed by thorough mixing and a 24-h standing period to allow for layer separation. The upper n-hexane layer was collected, leaving a residue in the aqueous fraction. Next, the same procedure was repeated with chloroform, again in three 4800 mL additions, to yield a chloroform layer. Finally, ethyl acetate was used in three 4800 mL increments under the same conditions, yielding an ethyl acetate layer. Each layer was evaporated under reduced pressure at 39 °C and prepared for MTT and DCF-DA assays to determine the most potent fraction.

After liquid–liquid fractionation, to further purify the bioactive fraction obtained from preliminary fractionation, open column chromatography was performed using silica gel as the stationary phase. Briefly, the silica gel (328.25 g) was first activated by drying at 100 °C for at least two hours and then packed into a glass column under gentle tapping to ensure uniform packing. The chosen fraction from the liquid–liquid fractionation, based on the MTT and DCF-DA assays, was dissolved in a minimal volume of the initial mobile phase and carefully loaded onto the top of the silica bed. After, an elution gradient was established with a binary solvent system (i.e., chloroform and ethanol); a stepwise gradient was initiated from 100:0 (*v*/*v*) chloroform–ethanol to 90:10, 80:20, 70:30, and so on, until reaching 0:100. Eluent was passed through the column under gravity and fractions (790 mL each) were collected sequentially. A total of 11 fractions were obtained, evaporated, and then subjected to the MTT and DCF-DA assays.

Preparative TLC was performed to separate the active compound from the selected fraction, which represented the highest attenuating effects against oxidative stress among the fractions. Briefly, a selected fraction was dissolved in absolute ethanol (300 mg/mL), then spotted on a silica gel 30 cm × 30 cm TLC plate (Merck; Darmstadt, Germany). One microliter of spotted sample on the plate was developed in the solvent mixture of chloroform and ethanol (55:45 mL) and visualized using an ultraviolet lamp (245 and 365 nm). After completing the development of TLC bands, each band was scraped and extracted with ethanol for the measurements of protective effects (i.e., MTT and DCF-DA assays).

### 3.7. High-Pressure Liquid Chromatography (HPLC) Analysis

In order to isolate the active compound from the fraction of preparative TLC, an HPLC system coupled with a YL9160 PDA detector was utilized. A Capcell Pak C18 MG S5 column (reverse phase column, size: 4.6 × 250 mm) was employed at 23 °C, with a flow rate of 1 mL/min and wavelength of 200–800 nm. Separation was conducted with a linear gradient of 0–100% ethanol over 90 min. The sample was dissolved in HPLC-grade ethanol at the concentration of 1 mg/mL, and the injection volume was 20 μL.

### 3.8. Elucidation of Active Component Structure: Gas Chromatography (GC)–Mass Spectrometry (MS) and ^1^H and ^13^C-Nuclear Magnetic Resonance (NMR) Analyses

GC-MS analysis was performed using an Agilent 6890 Plus gas chromatography system, equipped with a 5977 N mass selective detector quadruple mass spectrometer system (Palo Alto, CA, USA). The MS capillary column (30 mm × 0.25 mm, 0.25 μm film thickness, 5% diphenyl–95% dimethylsiloxane phase) was obtained from J&W Scientific (Folsom, CA, USA). The temperatures of the GC injection port and MS interface were run in the electron impact ionization (EI) mode, with electron energy at 79 eV. The mass spectrometer was operated in full scan mode between 50 and 700 amu. In order to identify active constituents, the Wiley 7 N spectra database was used. Subsequently, the ^13^C and ^1^H-NMR were carried out and recorded on a high-resolution NMR (Avance-600, Bruker; Karlsruhe, Germany) spectrometer operating at 700 MHz and 25 °C. The sample was dissolved in methyl-d3 alcohol-dl.

### 3.9. In Vivo Experiment I and II: Mouse Intervention Studies Using RR Roots Extract and Oleamide Therein

ICR (Institute of Cancer Research) male mice (five weeks old) were obtained from Daehan Biolink (*n* = 7–8/group; Chungnam, Republic of Korea). The mice were housed nine per cage in a room maintained with a 12-hr light–dark cycle, 55% humidity, and 23–25 °C temperature. For the crude extract intervention (i.e., in vivo experiment I), RR roots extract was mixed in a commercial diet at a concentration of 400, 800, and 1200 mg/kg body weight. For the oleamide intervention (i.e., in vivo experiment II), the compound was mixed with the commercial diet at a concentration of 10, 20, and 40 mg/kg body weight. The ICR mice were allowed free access to feed and water for 3 weeks, then administrated Aβ via intracerebroventricular (ICV) injection. Aβ was dissolved in 0.85% (*w*/*v*) of sodium chloride solution, and the injection volume was 5 μL (i.e., 410 pmol/mouse). A Hamilton micro-syringe fitted with a 26-gauge needle was inserted to a depth of 2.5 mm. The animal intervention study protocol was approved by the Korea University IACUC review (KUIACUC-2017-142).

### 3.10. Y-Maze Test: Assessment of Immediate Working Memory

Spontaneous alternation behavior in a Y-maze was used to evaluate immediate working memory. The test was performed three days after intracerebroventricular (ICV) injection of Aβ_1-42_ to allow for the development of cognitive impairment [21]. The Y-maze, constructed of black-painted plastic, consisted of three arms each measuring 33 cm in length, 15 cm in height, and 10 cm in width, arranged at equal angles. Mice were placed at the end of one arm and allowed to explore freely for 8 min. An arm entry was counted only when the mouse’s hind paws were fully within that arm. An alternation was defined as consecutive entries into all three arms in overlapping triplet sets. The percentage of alternation was calculated as [actual alternations/(total arm entries − 2)] × 100.

### 3.11. Passive Avoidance Test: Assessment of Aversion Learning Ability

A passive avoidance test was performed to assess the aversion learning ability of mice as reported elsewhere [20]. A passive avoidance apparatus with two compartments—a lighted side and a dark side—was used. During the training trial, each mouse was placed in the illuminated compartment. Upon entering the dark compartment, the mouse received an unavoidable electric shock (0.5 mA for 1 s). One day later, a retention trial was conducted: the mouse was again placed in the lighted compartment, and the time taken to re-enter the dark compartment (step-through latency) was recorded, with a maximum limit of 300 s.

### 3.12. Evaluation of Acute Toxicity of RR Root Extract and Oleamide In Vivo

After the behavior tests, the acute toxicity of the samples was measured using the serum transaminase reagents kit (AM 101-K, Asan Pharmaceutical; Seoul, Republic of Korea) according to the manufacturer’s instructions.

### 3.13. Assessment of Lipid Peroxidation Using the ICR Mice Brain

Malondialdehyde (MDA) levels in brain homogenates were quantified using a thiobarbituric acid (TBA)–based assay. Briefly, 80 μL of each homogenate was mixed with 1% (*v*/*v*) phosphoric acid and 160 μL of 0.67% (*w*/*v*) TBA. The mixture was then heated at 95 °C for 45 min. After cooling to room temperature, n-butanol was added to extract the resulting colored complex, and absorbance was measured at 532 nm using a UV-1601 spectrophotometer (Shimadzu; Kyoto, Japan). Protein content in each sample was determined by the Bradford method (Bio-Rad; Hercules, CA, USA), and final MDA levels were expressed as nanomoles per milligram of protein.

### 3.14. Assessment of Acetylcholine (ACh) Levels in the ICR Mice Brain

The ACh content was measured using the Hestrin method as described previously [22]. Brain homogenate (1 mL) was mixed with 2 mL of alkaline hydroxylamine reagent. After 1 minute, 1 mL of hydrochloride solution and 1 mL of iron solution were added. The density of the purple–brown color was determined (540 nm).

### 3.15. Statistical Analysis

The data are presented as mean ± standard deviation. The normality of distribution was assessed for each dataset using the Shapiro–Wilk test. If the data were normally distributed (*p* > 0.05), a one-way ANOVA followed by Dunnett’s post hoc test was used to compare the experimental groups. If normality was not satisfied (*p* ≤ 0.05), the Kruskal–Wallis test followed by Dunn’s multiple comparisons test was applied. A *p*-value of 0.05 or less was considered statistically significant, and GraphPad Prism (Ver. 7.00) was used for the analyses (GraphPad Software; San Diego, CA, USA).

## 4. Conclusions

To summarize, our study provides compelling evidence that RR root extract and its bioactive constituent, oleamide, exhibit significant neuroprotective effects against Aβ-induced toxicity. By employing a multi-step biochemical and analytical approach, oleamide was identified as the key neuroactive compound within RR root extract. Furthermore, behavioral improvements observed in the Y-maze and passive avoidance tests suggest that oleamide mitigates Aβ-induced cognitive impairments in a dose-dependent manner. Importantly, the absence of overt toxicity at neuroprotective doses strengthens the case for oleamide as a viable candidate for further applications in humans. Future studies should focus on elucidating the precise molecular pathways underlying oleamide’s neuroprotective actions, investigating its long-term efficacy in chronic AD models, and assessing potential synergistic effects when combined with existing Aβ-targeting or anti-inflammatory therapies.

## Figures and Tables

**Table 1:** Protective effects of natural plant extracts against oxidative stress.

Scientific Name	Protective Effect (%) ^a^	Cell Viability (%) ^b^
*Euryale ferox Salisb.*	77.7	90.8
*Tussilago farfara* L.	73.7	67.1
*Chrysosplenium ramosum maxim*	59.8	48.4
*Allium ascalonicum* L.	60.1	50.7
*Carya pecan*	78.5	78.5
*Brassica oleracea var. acephala*	31.6	46.0
*Ilex paraguayensis*	85.0	70.1
*Rosa rugosa (roots)*	91.5	121.4
*Brassica juncea var. integrifolia*	44.2	54.9
*Perilla frutescens var. japonica (Hassk.) Hara*	35.5	46.4
*Chrysanthemum coronarium var. spatiosum*	2.5	41.6
*Prunus salicina*	33.6	48.1
*Ocimum basilicum*	21.3	45.3
*Fraxinus rhynchophylla HANCE*	31.3	44.7
*Pimpinella brachycarpa*	16.5	38.0
*Spinacia oleracea*	50.8	42.7
*Phaseolus radiatus*	55.2	46.0
*Carthamus tinctorius*	31.9	45.4
*Ulmus pumila* L.	85.3	64.1
*Adenophora triphylla var. janponica Hara*	38.7	45.5
*Cuscuta japonica Choisy*	70.0	49.4
*Brassica napus*	49.0	51.9
*Acorus gramineus*	31.5	46.1
*Acorus calamus var. angustatus*	49.8	41.3
*Cucurbita* spp.	11.3	37.8
*Cichorium intybus*	53.6	38.6

^a^ Protective effect (%) = 100 − [(DCF formation of sample group with oxidative stress − DCF formation of negative (NEG) group without oxidative stress) × 100/(DCF formation of positive (POS) group with oxidative stress − DCF formation of NEG group without oxidative stress)]. ^b^ Cell viability = formazan formation of sample group with oxidative stress × 100/formazan formation of NEG group without oxidative stress. Each extract was reconstituted in 5% dimethyl sulfoxide (*w*/*v*) to a final concentration of 1 mg/mL for screening assays. Abbreviations: NEG, untreated group; POS, hydrogen peroxide (200 μM) treated group.

## Data Availability

Data is contained within the article.

## References

[1] Nakabayashi J., Yoshimura M., Morishima-Kawashima M., Funato H., Miyakawa T., Yamazaki T., Ihara Y. (1998). Amyloid (B-protein (Aß) Accumulation in the Putamen and Mammillary Body during Aging and in Alzheimer Disease. J. Neuropathol. Exp. Neurol..

[2] Dai H., Hu M., Li Q., Zhang L., Zhu D., Li Q., Li S., Liu T., Li X. (2023). Identification of Causal Relationship Between Amyloid-Beta Accumulation and Alzheimer’s Disease Progression via Counterfactual Inference.

[3] Aschenbrenner D.S. (2021). Rn Controversial Approval of New Drug to Treat Alzheimer’s Disease. Am. J. Nurs..

[4] Schelke M.W., Hackett K., Chen J.L., Shih C., Shum J., Montgomery M.E., Chiang G.C., Berkowitz C., Seifan A., Krikorian R. (2016). Nutritional interventions for Alzheimer’s prevention: A clinical precision medicine approach. Ann. N. Y. Acad. Sci..

[5] Kim J.K., Choi S.J., Cho H.Y., Hwang H.-J., Kim Y.J., Lim S.T., Kim C.-J., Kim H.K., Peterson S., Shin D.-H. (2010). Protective Effects of Kaempferol (3,4′,5,7-tetrahydroxyflavone) against Amyloid Beta Peptide (Aβ)-Induced Neurotoxicity in ICR Mice. Biosci. Biotechnol. Biochem..

[6] El Gaamouch F., Chen F., Ho L., Lin H.Y., Yuan C., Wong J., Wang J. (2022). Benefits of dietary polyphenols in Alzheimer’s disease. Front. Aging Neurosci..

[7] Nam M.-H., Lee H.-S., Hong C.-O., Koo Y.-C., Seomun Y., Lee K.-W. (2010). Preventive effects of *Rosa rugosa* root extract on advanced glycation end product-induced endothelial dysfunction. Korean J. Food Sci. Technol..

[8] Choi D.H., Han J.H., Hong M., Lee S.Y., Lee S.U., Kwon T.H. (2021). Antioxidant and lipid-reducing effects of *Rosa rugosa* root extract in 3T3-L1 cell. Food Sci. Biotechnol..

[9] Lu J., Wang C. (2018). Medicinal Components and Pharmacological Effects of *Rosa rugosa*. Rec. Nat. Prod..

[10] Park B.-J. (2008). Isolation of main component and antioxidant activities on the stem and root of *Rosa rugosa*. Korean J. Plant Res..

[11] Na J.-R., Oh D.-R., Han S., Kim Y.-J., Choi E., Bae D., Oh D.H., Lee Y.-H., Kim S., Jun W. (2016). Antistress Effects of *Rosa rugosa* Thunb. on Total Sleep Deprivation–Induced Anxiety-Like Behavior and Cognitive Dysfunction in Rat: Possible Mechanism of Action of 5-HT_6_ Receptor Antagonist. J. Med. Food.

[12] Park J.C., Kim S.C., Hur J.M., Choi S.H., Lee K.Y., Choi J.W. (2004). Anti-Hepatotoxic Effects of *Rosa rugosa* Root and Its Compound, Rosamultin, in Rats Intoxicated with Bromobenzene. J. Med. Food.

[13] Hestrin S. (1949). The Reaction of Acetylcholine and Other Carboxylic Acid Derivatives with Hydroxylamine, and its Analytical Application. J. Biol. Chem..

[14] Akanmu M.A., Adeosun S.O., Ilesanmi O.R. (2007). Neuropharmacological effects of oleamide in male and female mice. Behav. Brain Res..

[15] Pedersen W.A., Kloczewiak M.A., Blusztajn J.K. (1996). Amyloid beta-protein reduces acetylcholine synthesis in a cell line derived from cholinergic neurons of the basal forebrain. Proc. Natl. Acad. Sci. USA.

[16] Heo H.-J., Park Y.-J., Suh Y.-M., Choi S.-J., Kim M.-J., Cho H.-Y., Chang Y.-J., Hong B., Kim H.-K., Kim E. (2003). Effects of Oleamide on Choline Acetyltransferase and Cognitive Activities. Biosci. Biotechnol. Biochem..

[17] Ano Y., Ozawa M., Kutsukake T., Sugiyama S., Uchida K., Yoshida A., Nakayama H. (2015). Preventive Effects of a Fermented Dairy Product against Alzheimer’s Disease and Identification of a Novel Oleamide with Enhanced Microglial Phagocytosis and Anti-Inflammatory Activity. PLoS ONE.

[18] Kita M., Ano Y., Inoue A., Aoki J. (2019). Identification of P2Y receptors involved in oleamide-suppressing inflammatory responses in murine microglia and human dendritic cells. Sci. Rep..

[19] Pahan K. (2020). Stimulation of ADAM10 and decrease in plaques by a sleep-inducing supplement. Alzheimer’s Dement..

[20] Zhang N., Xu H., Wang Y., Yao Y., Liu G., Lei X., Sun H., Wu X., Li J. (2020). Protective mechanism of kaempferol against Aβ25-35-mediated apoptosis of pheochromocytoma (PC-12) cells through the ER/ERK/MAPK signalling pathway. Arch. Med. Sci..

[21] Kim J.K., Choi S.J., Bae H., Kim C.R., Cho H.-Y., Kim Y.J., Lim S.T., Kim C.-J., Kim H.K., Peterson S. (2011). Effects of Methoxsalen from *Poncirus trifoliata* on Acetylcholinesterase and Trimethyltin-Induced Learning and Memory Impairment. Biosci. Biotechnol. Biochem..

[22] Chang S.W., Du Y.E., Qi Y., Lee J.S., Goo N., Koo B.K., Bae H.J., Ryu J.H., Jang D.S. (2019). New Depsides and Neuroactive Phenolic Glucosides from the Flower Buds of Rugosa Rose (*Rosa rugosa*). J. Agric. Food Chem..

